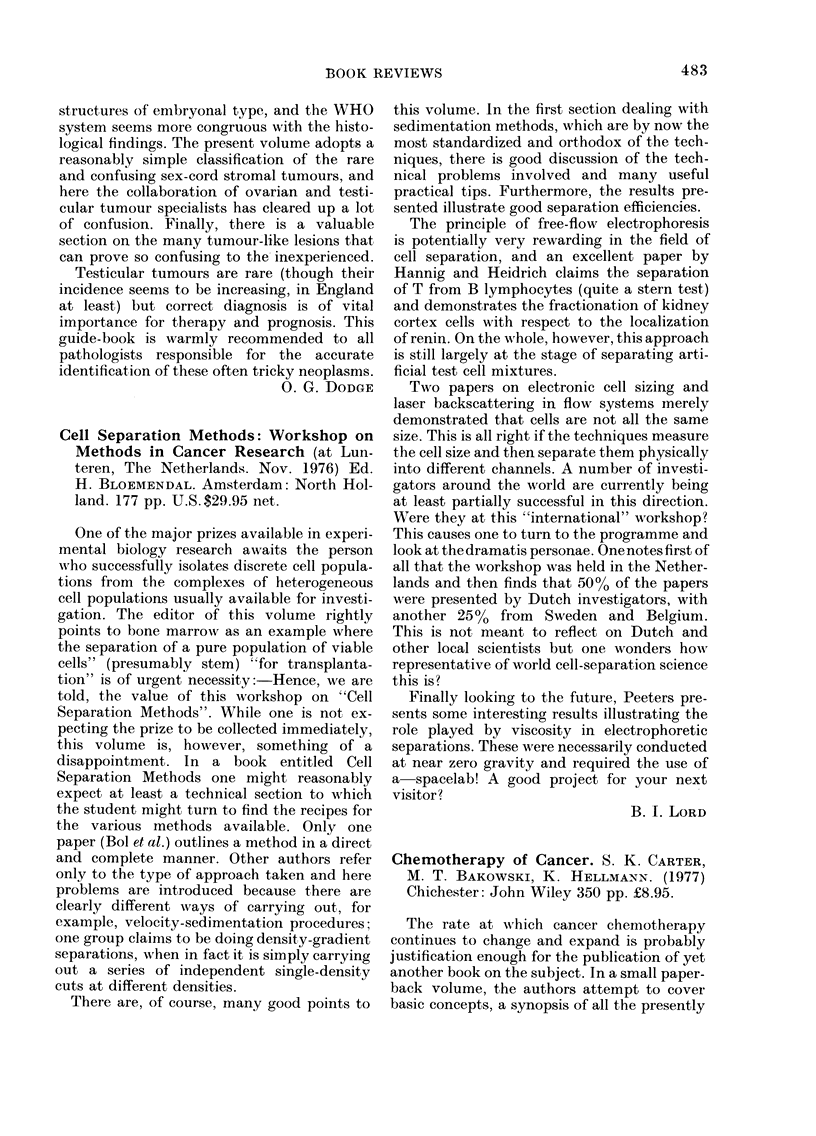# Cell Separation Methods: Workshop on Methods in Cancer Research

**Published:** 1978-03

**Authors:** B. I. Lord


**Cell Separation Methods: Workshop on Methods in Cancer Research** (at Lunteren, The Netherlands. Nov. 1976) Ed. H. BLOEMENDAL. Amsterdam: North Holland. 177 pp. U.S.$29.95 net.

One of the major prizes available in experimental biology research awaits the person who successfully isolates discrete cell populations from the complexes of heterogeneous cell populations usually available for investigation. The editor of this volume rightly points to bone marrow as an example where the separation of a pure population of viable cells" (presumably stem) "for transplantation" is of urgent necessity:—Hence, we are told, the value of this workshop on "Cell Separation Methods". While one is not expecting the prize to be collected immediately, this volume is, however, something of a disappointment. In a book entitled Cell Separation Methods one might reasonably expect at least a technical section to which the student might turn to find the recipes for the various methods available. Only one paper (Bol *et al.*) outlines a method in a direct and complete manner. Other authors refer only to the type of approach taken and here problems are introduced because there are clearly different ways of carrying out, for example, velocity-sedimentation procedures; one group claims to be doing density-gradient separations, when in fact it is simply carrying out a series of independent single-density cuts at different densities.

There are, of course, many good points to this volume. In the first section dealing with sedimentation methods, which are by now the most standardized and orthodox of the techniques, there is good discussion of the technical problems involved and many useful practical tips. Furthermore, the results presented illustrate good separation efficiencies.

The principle of free-flow electrophoresis is potentially very rewarding in the field of cell separation, and an excellent paper by Hannig and Heidrich claims the separation of T from B lymphocytes (quite a stern test) and demonstrates the fractionation of kidney cortex cells with respect to the localization of renin. On the whole, however, this approach is still largely at the stage of separating artificial test cell mixtures.

Two papers on electronic cell sizing and laser backscattering in flow systems merely demonstrated that cells are not all the same size. This is all right if the techniques measure the cell size and then separate them physically into different channels. A number of investigators around the world are currently being at least partially successful in this direction. Were they at this "international" workshop? This causes one to turn to the programme and look at the dramatis personae. One notes first of all that the workshop was held in the Netherlands and then finds that 50% of the papers were presented by Dutch investigators, with another 25% from Sweden and Belgium. This is not meant to reflect on Dutch and other local scientists but one wonders how representative of world cell-separation science this is?

Finally looking to the future, Peeters presents some interesting results illustrating the role played by viscosity in electrophoretic separations. These were necessarily conducted at near zero gravity and required the use of a—spacelab! A good project for your next visitor?

B. I. LORD